# Lower accuracy of cytological screening for high-grade squamous intraepithelial neoplasia in women over 50 years of age in Japan

**DOI:** 10.1007/s10147-021-02065-w

**Published:** 2021-12-06

**Authors:** Michiyasu Miki, Yoshihiro Imaeda, Hiroshi Takahashi, Aya Iwata, Tetsuya Tsukamoto, Hiroyuki Nomura, Kiriko Kotani, Takeji Mitani, Ryoko Ichikawa, Takuma Fujii

**Affiliations:** 1 Department of Obstetrics and Gynecology, School of Medicine, Fujita Health University, 1-98, Dengakugakubo, Kutsukakecho, Toyoake, Aichi 470-1192 Japan; 2 Department of Pathology, School of Medicine, Fujita Health University, Toyoake, Aichi Japan; 3 Division of Medical Statistics, School of Medicine, Fujita Health University, Toyoake, Aichi Japan

**Keywords:** Cervical cancer, Screening, Cytology, Aging, Atrophy

## Abstract

**Background:**

As the population ages in developed countries, the number of Pap smears for cervical cancer screening of older women is increasing. There is concern that cervical atrophy may cause misinterpretation of results for this segment of the population. The present study evaluated the accuracy of screening for high-grade intraepithelial lesions (HSILs) in women younger or older than 50 years, to determine whether aging affects cytological interpretation.

**Methods:**

Patients with HSIL cytology (*N* = 1565) were dichotomized into those aged 20–49 years or aged ≥ 50 years. Association between histology results and age was examined. Pearson’s chi-squared test and Cochran-Armitage trend test were used for statistical analysis.

**Results:**

The positive predictive value (PPV) for cervical intraepithelial neoplasia (CIN)2 and worse was 65.2% (62/95) in older women but 87.3% (482/552) in younger women (*p* < 0.001). Older patients had a significantly lower PPV (*p* = 1.69 × 10^–8^). Separately analyzing chronic cervicitis, CIN1 and overt cancer grouped together, compared with another group composed of CIN2 and CIN3, we found that the PPV for CIN2 and CIN3 was lower in older than in younger women [44.2% (42/95)-vs-82.4% (455/552), *p* < 0.001], respectively.

**Conclusions:**

HSILs are associated with a wide range of disease categories as age increases, and the accuracy of HSIL interpretation is lower in older women.

**Supplementary Information:**

The online version contains supplementary material available at 10.1007/s10147-021-02065-w.

## Introduction

Cervical cancer is the fourth most common cancer in women worldwide [1]. In developed countries, Pap smears are used for cervical cancer screening; the Bethesda system is widely accepted for their interpretation. Primary cytological screening is scheduled until the age of 65 years in USA [2], whereas it has no age limit in Japan. More than 30% of Japan’s population, and 25% of Europe’s population, but merely 12% of Latin America and Caribbean populations are ≥ 60 years of age [3]. Despite these regional disparities in age distribution, issues related to aging are intensifying worldwide. Japan is the most aged society worldwide at the moment. One issue to be confronted by the healthcare system is therefore the increasing number of Pap smears from screening older women. Aging greatly affects cervical cytology, which cervical atrophy caused by estrogen depletion may cause results to be misinterpreted [4]. Most women with cytological results showing high-grade intraepithelial lesions (HSILs) according to the Bethesda system will have biopsy-confirmed cervical intraepithelial neoplasia (CIN)2 or CIN3 identified at the time of colposcopy. Aging may cause misinterpretation of HSILs resulting in unnecessary biopsies or anxiety in healthy women. Additionally, atypical cells that originate from cancer may be underestimated in older women, causing their lesions to be classified as HSILs. We thus focused on the HSIL category rather than other abnormalities, such as atypical squamous cells of undetermined significance [ASC-US], low-grade squamous intraepithelial lesions [LSIL], or atypical squamous cells—cannot exclude high-grade squamous intraepithelial lesions [ASC-H] because a large number of final histological results could be obtained for HSIL, followed by biopsies. To the best of our knowledge, the previous studies on discrepant age-related results focusing on the interpretation of Pap smears are sparse [5]. Therefore, the present study aimed to evaluate cytology results from younger and older women with HSIL who were treated in our institution, and to determine how aging affects their interpretation.

## Materials and methods

**Fig. 1:**
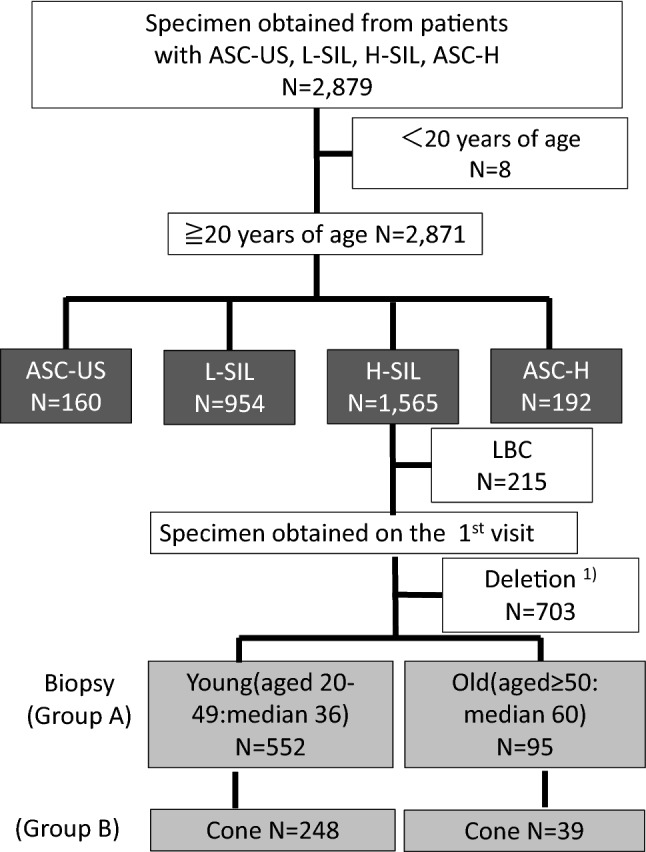
Flow chart of eligible patients. Patients were enrolled from April 2009 to March 2016 in our institution. For the interpretation of HSILs, 647 patients with colposcopy-directed punch biopsies were enrolled in Group A. Of those, 286 patients underwent cone resection (Group B). Details are provided in the Materials and Methods. 1) Specimen provided on the first visit was used for the study if multiple specimens were collected in follow-up visits. Multiple specimens from the same patients were excluded

We extracted information from the medical records of 2879 patients who visited our clinic between April 2009 and March 2016, with respect to cytological abnormalities, such as ASC-US, LSIL, HSILs, and ASC-H, and then further investigated those patients who had been diagnosed with HSIL (*N* = 1565) by cytology (Fig. 1). The patient population consisted of asymptomatic women and those who were being followed up for previous atypical smears or treatment of earlier gynecological malignancies.

Exfoliated cervical cells for conventional Pap smears were collected with a cervix brush or spatula, and fixed in ethanol at the time of collection. We excluded patients aged < 20 years, or whose specimens were obtained by liquid-based cytology (LBC). Median age of menopause in Japanese women is 50.5 years. Therefore, patients were dichotomized into younger (20–49 years) and older (≥ 50 years) ages (i.e., before/after median menopause age). The younger group had a median age of 36 years and the older 60 years. The histological results included chronic cervicitis, CIN1, CIN2, CIN3, and cancer. Patients were also classified into two other groups as follows: Group A (*N* = 647) consisted of patients who had one result at the first visit during this period. Results of repetitive examinations from the same patients during the observation period were excluded, so one histological result for each patient was analyzed. The median number of colposcopy-directed punch biopsies was two (range 1–5). There was no difference in the number of biopsies between the younger and older group. The histology results obtained from cone resection were extracted from Group A to constitute Group B (*N* = 287). The histology results of biopsies obtained from older women were particularly limited because worse lesions might have been hidden inside the transformation zone. In instances where patients underwent biopsies followed by cone resection, the final histology was determined as the highest-grade result.

### Statistical analysis

Performance of cytology results was expressed in terms of positive predictive value (PPV). Pearson’s chi-squared test was used to determine the association between age and disease category. A significance level of 0.05 was used for all statistical tests and two-tailed tests were applied. Cochran-Armitage trend test was used to determine the association between age and PPV. All statistical analyses were performed with SPSS version 22 (IBM Corp., Armonk, NY, USA).

## Results

### Discrepancy of histology results between younger and older patients

**Fig. 2:**
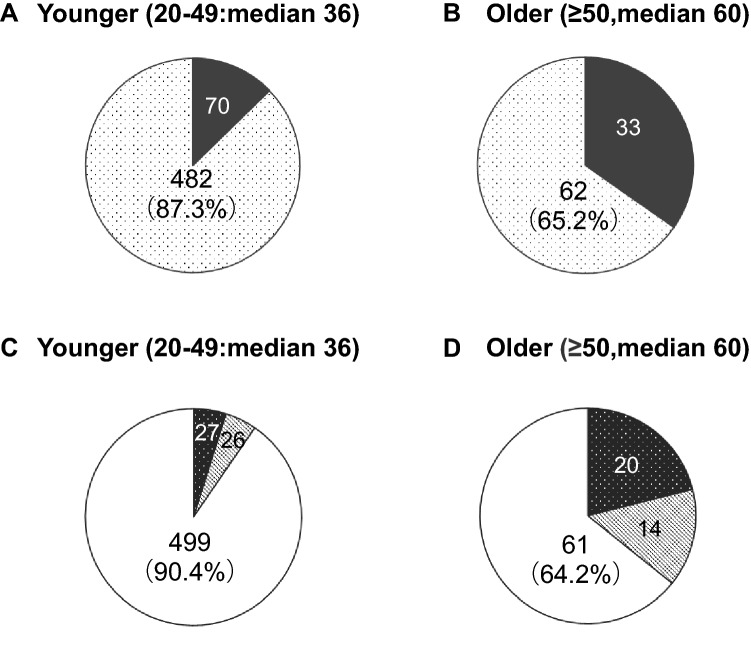
Distribution of final histology results between younger (median 36 years of age) and older women (60 of age) in HSIL. **A**: women aged 20–49 classified into chronic cervicitis + CIN1 (black) and CIN2 + (dot), B: women aged ≥ 50 classified into chronic cervicitis + CIN1 (black) and CIN2 + (dot). There was a significant difference (*p* < 0.001) between **A** and **B**. **C**: women aged 20–49 classified into Cancer (black dot), chronic cervicitis (gray) and CIN1-3 (white), **B**: women aged ≥ 50 classified into Cancer (black dot), chronic cervicitis (gray) and CIN1-3 (white). There was a significant difference (*p* < 0.001) between **C** and **D**

For statistical analysis, histological results were grouped as described below. Comparing the patients with CIN2 and worse (CIN2 +), we found that PPV for this group was significantly lower (*p* < 0.001) in older (65.2%, 62/95) than younger (87.3%, 482/552) patients, as shown in Table 1 and Fig. 2. With increasing age of the patients, PPV was significantly decreased (*p* = 1.69 × 10^–8^). Because we focused on the detection rate for CINs, we stratified the patients into two groups, one with chronic cervicitis and overt cancer and the other with CIN1-3. We found that PPV for the CIN1–3 group was significantly lower (*p* < 0.001) in older (64.2%, 61/95) than younger (90.4%, 499/552) patients. When comparing groups containing chronic cervicitis, CIN1 and overt cancer with CIN2 and CIN3 patients, we found that PPV for the CIN2/CIN3 group was also significantly lower (*p* < 0.001) in older (44.2%, 42/95) than younger (82.4%, 455/552) patients. Notably, the HSIL category in the older group included a significantly broader range of benign lesions, premalignant lesions and invasive cancer.

**Table 1:** Association between age group and final histology results

Histology	Young (median 36)				Old (median 60)					Total
	20–29	30–39	40–49	Subtotal	50–59	60–69	70–79	80-	Subtotal	
Chronic cervicitis	2	10	14	26	6	3	4	1	14	40
CIN1	7	16	21	44	12	4	3	0	19	63
CIN2	31	78	54	163	8	6	0	1	15	178
CIN3	57	146	89	292	12	9	5	1	27	319
Cancer	3	8	16	27	9	7	4	0	20	47
Total	100	258	194	552	47	29	16	3	95	647
PPV (CIN2 +)	91%	90%	82%	87.3%	62%	76%	56%	67%	65.2%	

The final histology obtained by biopsy or cone resection were determined by the high-grade result

### Comparison of histology results between biopsy and cone resection

We compared the histology results of biopsies and cone resections. Of 647 patients (Group A) who had biopsies, 287 underwent cone resection, as shown in Fig. 1. Of the cases undergoing cone resection with therapeutic purpose, the majority (86.4%, 248/287) were younger women, because 202 cases were younger women diagnosed with CIN3 (Table 2). Histology results leading to a diagnosis of CIN2/3 by sampling method were from biopsy only (85.4%, 245/287), or biopsy followed by cone resection (85.0%, 244/287). There were no significantly discrepant results between colposcopy-directed punch biopsy and cone resection (*p* = 0.906) as a whole. However, the diagnosis in 9 of 248 patients (3.6%) and 5 of 39 patients (12.8%) with cancer were significantly up-graded in younger and older women, respectively (*p* = 0.013) (Table 2). In individual cases, an up-graded diagnosis was made following cone resection in 12.8% (5/39) of older women with chronic cervicitis or CIN1 by biopsy, significantly more than only 2.8% (7/248) of younger women (*p*=0.0037) (Table 3). In contrast, underdiagnosis on the basis of cone resection results was found in 5 patients possibly due to the small size of the lesion (Supplementary Table 1). This finding indicates that cone resection should be recommended because histology results by biopsy may lead to underdiagnosis in patients with HSIL cytology, especially in older women.

**Table 2:** Histology results by biopsy VS cone

Histology	All ages	Colposcopy-directed biopsy		All ages	Cone	
		Young (median 36)	Old (median 60)		Young (median 36)	Old (median 60)
Chronic cervicitis	13	10	3	4	2	2
CIN1	11	5	6	6	3	3
CIN2	50	44	6	25	21	4
CIN3	195	179	16	219	202	17
Cancer	18	10	8	32	19	13
Other^a^	0	0	0	1	1	0
Total	287	248	39	287	248	39

^a^lobular endocervical glandular hyperplasia

**Table 3:** Up-grade※ results in cone resection from biopsy between younger and older women

	Young	Old
	(median 36)	(median 60)
Rate of up-grade ( +)	7/248 (2.8%)	5/39 (12.8%)#

※Up-grade defines from CC and CIN1 to CIN3 and worse in the identical patients. #Statistically significant (*p* = 0.0037)

## Discussion and conclusions

**Fig. 3:**
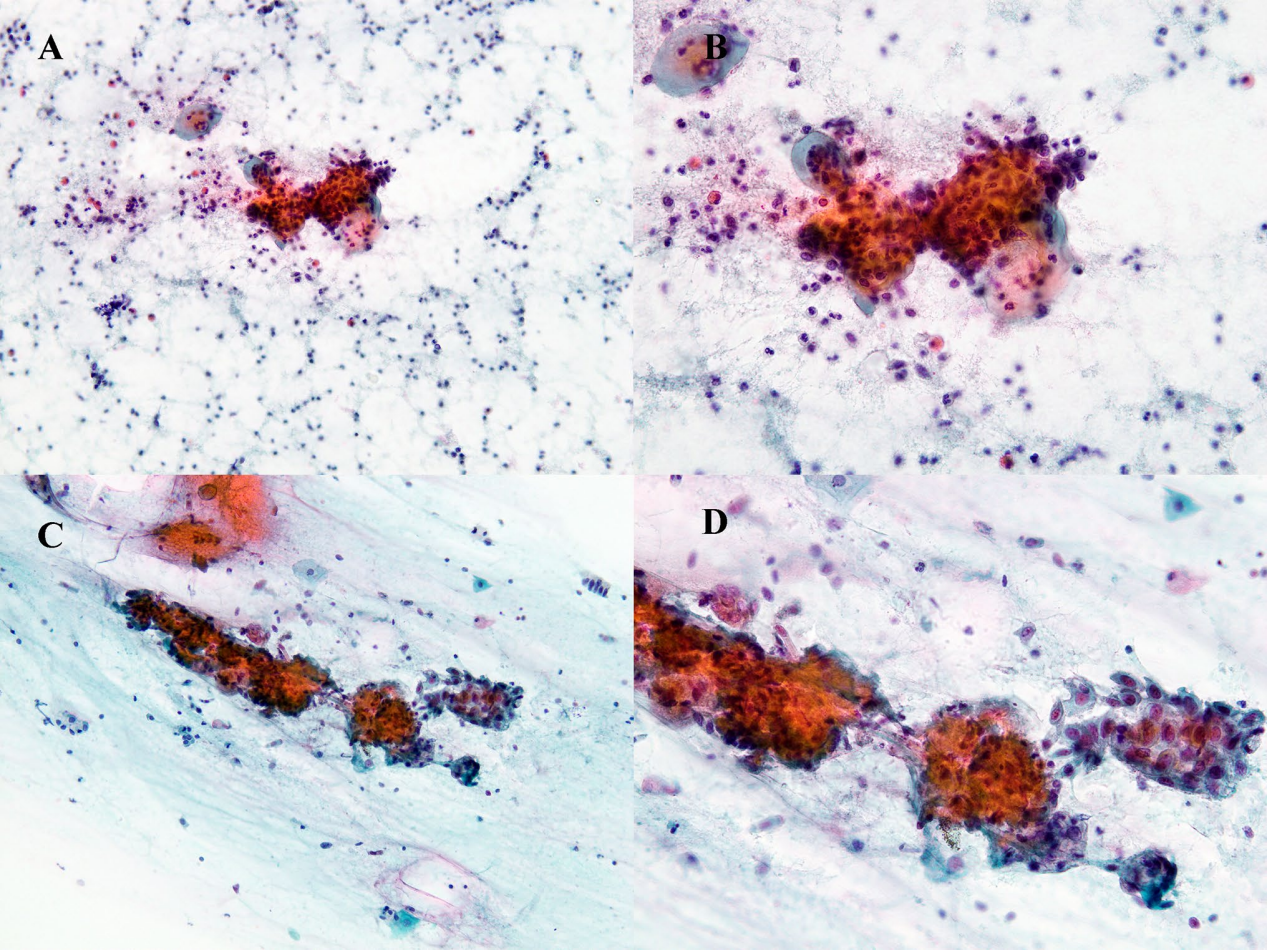
Interpretation of HSIL in older patients. Cytology from a patient with final histology results diagnosed as cervical cancer, **A**: low (× 10) and **B**: high (× 20) magnification. Cytology from a patient with final histology results indicating chronic cervicitis, **C**: low (× 10) and **D**: high (× 20) magnification. Atypical squamous cells in a syncytial pattern with enlarged hyperchromatic nuclei on a background of watery or inflammatory change, illustrating the difficulty of discriminating cancer from inflammatory change in both cytology specimens.

Interpretation of cytological results can be problematic if cellular atrophy is dominant in the specimens to be examined. Atrophy is a normal aging phenomenon with a wide spectrum of cellular changes and a variable amount of inflammation [6]. It is sometimes difficult to discriminate between an atrophic change and a dysplastic change with generalized nuclear enlargement (up to 3–5-times the area of an intermediate cell nucleus, and possibly with a slight increase in the nuclear/cytoplasmic ratio). Parabasal-type cells may have mild hyperchromasia and tend to have more elongated nuclei (Fig. 3). The vaginal dysbiome was observed in postmenopausal women [7], which also caused inflammation followed with hyperchromasia. Notably, air drying, which is a common practice with smear preparations of this type, especially in the specimens of older women, and may cause artefactual nuclear enlargement [6]. LBC would have been more advantageous for excluding such inappropriate specimens compared with conventional cytology [8].

Assessment of the HSIL category in cytology can be confirmed by colposcopy-directed punch biopsy or cone resection. Poomtavorn reported that in postmenopausal women, a broad range of diseases is categorized as HSIL [9]. However, whether results from younger and older patients differ significantly was unclear. We therefore examined histological results diverging between younger and older women. PPV for patients in the CIN2 + group was significantly lower in older than in younger women (Table 1 and Fig. 2). In the older patients, the HSIL category was not specific for high-grade CINs. Our findings show that HSILs in older women contained a broader range of histological results than in the younger patients.

The reason for these discrepancies between cytological and histological results may be related to sampling, screening and interpretation errors [10]. Atrophic changes cause screening and interpretation errors. Colposcopy-directed punch biopsies were often inappropriate due to the unsatisfactory findings for colposcopy in older women. The discrepancy rate (chronic cervicitis and CIN1) would be expected to be higher at older (33/95, 34.7%) than at younger (70/552, 12.7%) ages if the proportion of older women is higher in the tested population. When cancer screening is generally performed at 20 or 30 years of age, atrophy may not be an issue. In Japan, the proportion of women > 50 years of age made up 20% of the screened population [11]. In our study, 14.7% (95/647) were ≥ 50 years. This is not an exceptional situation in Japan. Women who received their first screening when they were already this age are not unusual.

This study has some limitations. It was a retrospective study that was conducted in one referral hospital. Histological and cytological results were obtained from medical records, and were not re-reviewed for this study. Cases were only evaluated by the conventional cytology. LBC was omitted from this analysis because of the small number of samples in the study. So far, conventional cytology was popular and LBC was not yet in widespread use in Japan [12]. Other confounding factors, including the clinical parameters, such as pregnancies, smoking habit, use of contraceptive pills, hormonal status, history of sexually transmitted infections, history of cervical abnormality, HPV infection status or menopausal status, were not considered in this analysis. Because the test population was not representative of the general population, sensitivity or specificity was not calculated. We did not evaluate the colposcopic findings, including transformation zone types and colposcopic interpretation of the worst lesions. We did not examine the use of vaginal estrogen before Pap test, even though atrophic cytology was significantly reduced in postmenopausal women who used a five-night regimen of vaginal estrogen before the Pap test [4].

In contrast, our study also has some strengths. In Japan, the Bethesda system for cervical cytology was adopted in 2008. The present study is the first to compare histological results in patients with HSILs the by conventional cytology in different age groups. The number of cases was sufficient for statistical analysis. We assumed that the colposcopy-directed punch biopsy was of limited value because inappropriate lesions might be taken. We thus adopted the histology results combined with cone biopsy as well as biopsy data.

In summary, older women with HSILs had a greater variety of lesions such as chronic cervicitis or cancer in addition to CINs, than did younger women. Interpretation of HSILs involves different disease categories complicated by aging. In Japan, a self-payment-free-coupon program for cervical cancer screening was conducted by a local government under the financial support from the Japanese national government. The target ages were 5-year intervals from 20 to 40 years. An approximately threefold increase was observed in the number of individuals undergoing Pap smear testing with this program [13]. If the target age is extended up to 50 years, more patients with cancer may be detected early. We assumed that interpretation of cervical cytology by the Bethesda system is best-suited for premenopausal women. It takes time approximately 10 years to develop cancer after the HPV infection. As elderly patients with cervical cancer have a poor prognosis [14, 15], we propose that cytology screening should be performed in women before menopause as well as at younger ages.

To minimize the performance of diagnostic cone resection, one option may be to use vaginal estrogen before repeating cytology tests. On the contrary, if gynecologists suspect cancer on the HSIL and colposcopic findings are inadequate, patients should undergo diagnostic cone resection without delay. We fear that a cervical cancer screening system based only on Pap smears may have decreasing utility in countries with increasingly aging populations. In this respect, screening by HPV testing or other auxiliary diagnostic tools, including p16 immunostaining [16] or other molecular testing, might be an alternative option.

## Supplementary Information

Below is the link to the electronic supplementary material.

Supplementary file1 (XLSX 12 kb)
